# Epiregulin expression and secretion is increased in castration-resistant prostate cancer

**DOI:** 10.3389/fonc.2023.1107021

**Published:** 2023-03-13

**Authors:** Marc Wiesehöfer, Benedikt Bernhard Gereon Raczinski, Caroline Wiesehöfer, Jaroslaw Thomas Dankert, Elena Dilara Czyrnik, Martin Spahn, Marianna Kruithof-de Julio, Gunther Wennemuth

**Affiliations:** ^1^ Department of Anatomy, University Duisburg-Essen, Essen, Germany; ^2^ Department of Urology, Lindenhofspital Bern, Bern, Switzerland; ^3^ Department of Urology, University Duisburg-Essen, Essen, Germany; ^4^ Department for BioMedical Research, Urology Research Laboratory, University of Bern, Bern, Switzerland; ^5^ Department of Urology, Inselspital, Bern University Hospital, Bern, Switzerland; ^6^ Department for BioMedical Research, Translation Organoid Research, University of Bern, Bern, Switzerland; ^7^ Bern Center for Precision Medicine, University of Bern and Inselspital, Bern, Switzerland

**Keywords:** prostate cancer, castration-resistant prostate cancer, enzalutamide-resistant LNCaP cells, epiregulin (EREG), epiregulin biosynthesis, miRNA-19

## Abstract

**Introduction:**

In prostate cancer, long-term treatment directed against androgens often leads to the development of metastatic castration-resistant prostate cancer, which is more aggressive and not curatively treatable. Androgen deprivation results in elevated epiregulin expression in LNCaP cells which is a ligand of EGFR. This study aims to reveal the expression and regulation of epiregulin in different prostate cancer stages enabling a more specific molecular characterization of different prostate carcinoma types.

**Methods:**

Five different prostate carcinoma cell lines were used to characterize the epiregulin expression on the RNA and protein levels. Epiregulin expression and its correlation with different patient conditions were further analyzed using clinical prostate cancer tissue samples. Additionally, the regulation of epiregulin biosynthesis was examined at transcriptional, post-transcriptional and release level.

**Results:**

An increased epiregulin secretion is detected in castration-resistant prostate cancer cell lines and prostate cancer tissue samples indicating a correlation of epiregulin expression with tumor recurrence, metastasis and increased grading. Analysis regarding the activity of different transcription factors suggests the involvement of SMAD2/3 in the regulation of epiregulin expression. In addition, miR-19a, -19b, and -20b are involved in post-transcriptional epiregulin regulation. The release of mature epiregulin occurs via proteolytic cleavage by ADAM17, MMP2, and MMP9 which are increased in castration-resistant prostate cancer cells.

**Discussion:**

The results demonstrate epiregulin regulation by different mechanism and suggest a potential role as a diagnostic tool to detect molecular alterations in prostate cancer progression. Additionally, although EGFR inhibitors false in prostate cancer, epiregulin could be a therapeutic target for patients with castration-resistant prostate cancer.

## 1 Introduction

Prostate cancer (PCa) is a global health burden and is one leading cause of cancer-related death. In 2020, more than 1.4 million new PCa cases and above 375,000 cancer-related deaths were recognized worldwide (1). The treatment of PCa strongly depends on the stage present at time of diagnosis. A systemic androgen deprivation therapy (ADT) is recommended for hormone-sensitive tumor recurrence due to the dependence on androgens. Anti-hormonal substances such as enzalutamide or abiraterone block ligand binding to receptor, nuclear import or DNA binding of AR (2, 3). But nevertheless 10 - 20% of PCa patients develop castration-resistant PCa (CRPC) within five years of follow up and more than 84% of patients have metastases present at the time of CRPC diagnosis (4). In addition to therapy resistance, CRPC cells induce cell proliferation, apoptosis resistance and dedifferentiation of surrounding cells due to secretion of growth factors and cytokines (5, 6).Therefore, mechanisms leading to CRPC including secondary alterations of the AR, AR bypass and lineage plasticity resulting in neuroendocrine PCa (NEPC) are widely discussed in PCa research. This switch towards a neuroendocrine phenotype is frequently treatment-induced (tNEPC) (7).

In a previous study, the expression of the growth factor epiregulin (EREG) was elevated in castration-sensitive LNCaP cells following androgen deprivation (8). EREG is a ligand of the Epidermal Growth Factor Receptor (EGFR) family. After ligand binding, EGFR family receptors activate important signaling pathways such as the PI3K/AKT or MAPK pathway, which stimulate cell proliferation and survival (9). Epiregulin is proteolytically cleaved by members of the A disintegrin and metalloproteases (ADAM) family and matrix metalloproteases (MMP) (10, 11). The mature EREG enters the extracellular space and activates the EGFR in an autocrine, juxtacrine, paracrine and endocrine manner (12). In most tumor entities, EREG is upregulated and associated with metastasis and poor prognosis (13). Although increased expression of EREG has already been demonstrated after androgen deprivation of PCa cells *in vitro* (14) and in a xenograft model (15), little is known regarding EREG deregulation during PCa progression. In this study, five cell lines representing different stages of PCa are used to investigate the presence of EREG at variant PCa stages *in vitro* and putative causes for elevated EREG secretion.

## 2 Materials and methods

### 2.1 Cell lines

LNCaP, 22Rv1 and DU145 cells were grown in RPMI 1640 (Thermo Fisher Scientific, Schwerte, Germany) supplemented with 10% heat inactivated FCS (Sigma-Aldrich, Hamburg, Germany), l‐Glutamin (1 mM), sodium pyruvate (1 mM), Penicillin (100 U/mL) and Streptomycin (100 μg/mL) (Thermo Fisher Scientific, Schwerte, Germany). PC3 cells were grown in 50% Ham’s F‐12K (Thermo Scientific, Schwerte, Germany) and 50% RPMI 1640 supplemented with 10% heat inactivated FCS, l‐Glutamin (1 mM), sodium pyruvate (1 mM), Penicillin (100 U/mL) and Streptomycin (100 μg/mL). LNCaP, DU145 and PC3 cells were purchased from ATCC/LGC Standards GmbH (Wesel, Germany). The last authentication for LNCaP and DU145 cells occurred in 2020 whereas PC3 cells were authenticated in 2021 by ATCC performing STR Profiling following ISO 9001:2008 and ISO/IEC 17025:2005 quality standards. All analyzed cell lines were similar to ATCC human cell lines. 22Rv1 cells were obtained in 2020 from Leibniz Institute DSMZ-German Collection of Microorganisms and Cell Cultures GmbH (Braunschweig, Germany). The implementation for mycoplasma testing is described elsewhere (16). All experiments were performed with mycoplasma-free cells. Activation of the TGF-β signaling pathway in DU145 cells was achieved by treating cells with TGF-β1 and TGF-β2 (ImmunoTools, Friesoythe, Germany). Cell culture images were acquired using a Nikon eclipse Ts2 microscope (Nikon, Düsseldorf, Germany) and IC Capture software (Imaging Source Europe GmbH, Bremen, Germany).

### 2.2 Generation of enzalutamide-resistant LNCaP (LNCaP_EnzR_) cells

LNCaP cells were cultured for four weeks in RPMI 1640 without phenol red (Thermo Fisher Scientific, Schwerte, Germany) containing 10% heat inactivated charcoal-stripped FCS (Sigma-Aldrich, Hamburg, Germany) (androgen-free), l‐Glutamin (1 mM), sodium pyruvate (1 mM), Penicillin (100 U/mL) and Streptomycin (100 μg/mL). After four weeks, 10 µM (final conc.) enzalutamide (TargetMol, Wellesley Hills, MA, US) was added to the differentiation medium and cells were cultured for five additional months. To characterize the alterations, cells were examined morphologically using a light microscope (2.1) and flow cytometry (2.5). In addition, marker gene expression of epithelial prostate cells as well as neuroendocrine cells were determined by qRT-PCR (2.4).

### 2.3 RNA extraction

Total and small RNA extraction from monolayer cells were performed with NucleoSpin miRNA, Mini kit for miRNA and RNA purification (Machery-Nagel, Düren, Germany) in accordance to manufacturer’s instructions.

### 2.4 Quantitative real-time PCR

mRNA was reversed transcribed using High‐Capacity cDNA Reverse transcription Kit (Applied Biosystems, Darmstadt, Germany) while miRNA was reverse transcribed by miScript II RT Kit (Qiagen, Hilden, Germany). QRT‐PCRs were performed using qTOWER³ (Analytik Jena, Jena, Germany), specific primers and 5x EvaGreen ^®^ QPCR‐Mix II (ROX) (Bio‐Budget, Krefeld, Germany). The thermal cycling conditions were as followed: 95°C for 15 min followed by 45 cycles of 95°C for 15 s, 56 - 58°C for 30 s and 72°C for 30 s. Melting curve analysis was performed for quality control. Evaluation of relative mRNA or miRNA expression was determined by ΔΔCt method using GAPDH (for mRNA) or 5.8S rRNA (for miRNA) as housekeeping genes. The qRT-PCR oligonucleotide sequences are shown in **Supplementary Table 1**.

### 2.5 Flow cytometry

Cells were detached and resuspended in flow cytometry buffer (2/3 (v/v) PBS and 1/3 (v/v) RPMI without additives). For immunostaining, 10 μg/mL α-EREG (goat, polyclonal, R&D Systems, Minneapolis, Minnesota, US) or α-TACE/ADAM17 (mouse, MM0561-8C13, Novus Biologicals, Wiesbaden, Germany) antibody was added to flow cytometry buffer and incubated for 1 h at 4°C. ChromPure goat or mouse IgG (Jackson ImmunoResearch, Cambridgeshire, UK) were used as controls for corresponding primary antibody. After three washing steps, cells were incubated for 1 h at 4°C with FITC-conjugated α-goat (donkey, polyclonal, Jackson ImmunoResearch, Cambridgeshire, UK) or α-mouse (goat, polyclonal, Jackson ImmunoResearch, Cambridgeshire, UK) antibody diluted 1:50 in flow cytometry buffer. Finally, cells were washed three times and resuspended in PI diluted 1:200 in DMEM without additives. The acquisition was performed by FACS Calibur System (Becton Dickinson, Franklin Lakes, NJ, USA) and analyzed by Cell Quest Pro™ Version 6 (Becton Dickinson, Franklin Lakes, NJ, USA). To determine the size and granularity of cells the forward (FSC) and sideward (SSC) scatter were used.

### 2.6 Enzyme-linked immunosorbent assay (ELISA)

The protein content of EREG in the cell culture supernatant was determined by sandwich ELISA using the Human Epiregulin DuoSet ELISA (R&D Systems, Minneapolis, Minnesota, US) according to the manufacturer’s instructions. Absorbance was measured using a CLARIO starPlus microplate reader (BMG Labtech, Ortenberg, Germany). The total amount of EREG was calculated using a standard with known concentrations. The protein content of MMP2 and MMP9 in the cell culture supernatant was determined by solid phase ELISA. The detailed procedure is described in the **Supplementary information**.

### 2.7 Human prostate specimens and tissue microarray (TMA)

PCa tissue was obtained from patients recruited from the EMPaCT tumor bank (European Multicenter Prostate Cancer Clinical and Translational Research Group) as described previously (17). The study was approved by the local ethics committee (KEK Bern no. 128/2015). Tissue micro arrays have been generated by multiple tumor samples derived from the index lesion and include more differentiated areas of each tumor and matched lymph node metastasis from previously untreated patients, and characterized for several tumor relevant genes (e.g. AR, PTEN, p53, MLH1, CD44, ALDH1, chromogranin A, and synaptophysin), the TMPRSS2-ERG gene fusion (18, 19).

### 2.8 Immunohistochemistry

Paraffin‐embedded tissues were deparaffinized with xylene and rehydrated in a descending alcohol series (100%, 96% and 70%). Slices were boiled in citrate buffer (pH = 6), blocked with 1% BSA in PBS (60 min, room temperature) and incubated overnight at 4°C in 0.5% BSA in PBS containing 10 μg/mL α-EREG (goat, polyclonal, R&D Systems, Minneapolis, Minnesota, US) antibody. After three washing steps with PBS, slices were incubated (1 h, room temperature) in 0.5% BSA in PBS with 1:200 diluted biotinylated α-goat (rabbit, polyclonal, Dako, Agilent Technologies, Waldbronn, Germany) antibody. After washing with PBS three times, signal enhancement was achieved by Vectastain^®^ (Linaris, Wertheim‐Bettingen, Germany) according to the manufacturer’s instructions. Immunoreaction was visualized with diaminobenzidine (DAB) (Sigma-Aldrich, Hamburg, Germany), followed by nuclear staining using hematoxylin solution (Thermo Fisher Scientific, Oberhausen, Germany). Finally, slices were dehydrated and mounted with Xylene Substitute Mountant (Thermo Fisher Scientific, Oberhausen, Germany). Slides were scanned using a Aperio ScanScope Slide Scanner (Leica Biosystems, Nußloch, Germany) and saved as ScanScope Virtual Slide (.svs) files.

### 2.9 Immunoreactive score (IRS)

The Immunoreactive score (IRS) was determined according to Remmele and Stegner (20). Two researchers independently evaluated the percentage of stained cells as well as the intensity of the staining and calculated the score. Finally, the mean value was calculated and the IRS was determined.

### 2.10 SDS-PAGE and western blotting

The procedures for SDS-PAGE, Western Blotting and Immunodetection is described elsewhere (13). Immunodetection of proteins was carried out using antibodies shown in **Supplementary Table 2**.

### 2.11 Target gene prediction

MiRNA target gene prediction was carried out using TargetScan Human Release 7.2 (21).

### 2.12 Plasmids

Nucleotides 3853 - 4363 of the EREG mRNA (accession number: NM_001432.3) were amplified from human gDNA by PCR and inserted into pMIR-RNL-TK reporter plasmid (Ambion, Kaufungen, Germany). Mutagenesis of the predicted target site seed sequences of reporter constructs were performed by site directed mutagenesis. The miRNA expression plasmids were generated by PCR amplification of nucleotides 91,350,658 - 91,351,156 of chromosome 13 (+) for miR-19a-3P and nucleotides 91,350,960 - 91,351,560 of chromosome 13 (+) for miR-19b-3P from human gDNA. Subsequently, the DNA fragments were inserted into the pSG5 vector (Agilent technologies, Ratingen, Germany). Expression plasmid for miR-20b is described elsewhere (8). The oligonucleotide sequences used for molecular cloning and site directed mutagenesis are shown in **Supplementary Table 3**.

### 2.13 Transfection

The procedure for transfection of eukaryotic cells is described elsewhere (16).

### 2.14 Dual‐luciferase assay

HEK293T cells were transfected with 0.8 μg of expression plasmid and 0.2 μg reporter plasmid. After 48 h luciferase reporter assays were performed using the Dual‐Luciferase Reporter Assay System in accordance to manufacturer’s instructions (Promega, Mannheim, Germany). Luminescence was subsequently determined using a Lucetta™ luminometer (Lonza, Cologne, Germany).

### 2.15 Data analysis and statistical methods

TMAs were visualized with SlideViewer (Sysmex Germany GmbH, Norderstedt, Germany). Densitometrical analysis of immunoblots were quantified by ImageJ 1.48v (National Institute of Health, Bethesda, USA). Graphical illustration and statistical evaluation was performed with GraphPad Prism 9 (Statcon GmbH, Witzenhausen, Germany). Two-sided Student’s t-test was performed to compare two data sets, while ordinary one-way ANOVA was used to compare more than two datasets. p-values of <0.05 were defined as significant.

## 3 Results

### 3.1 LNCaP_EnzR_ cells show characteristics of NE-like PCa cells

**Figure 1:**
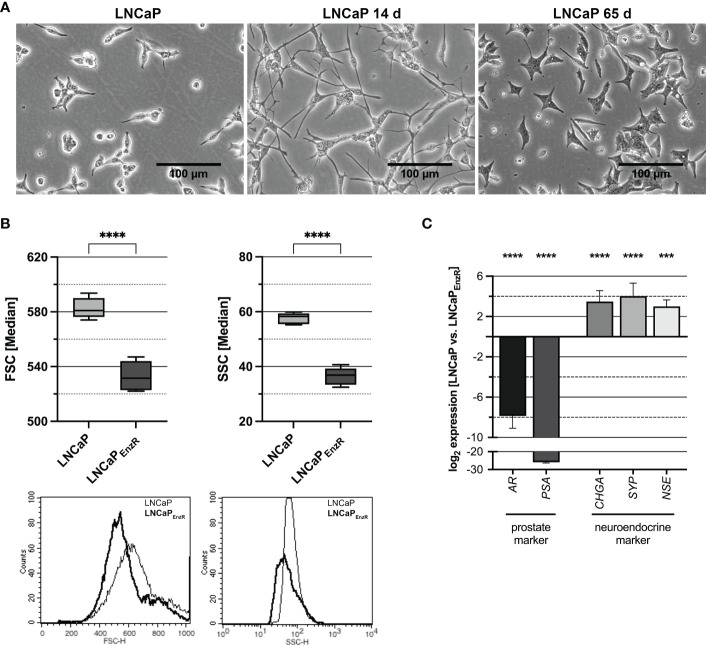
Morphology and marker gene expression of LNCaP cells after ADT and enzalutamide treatment. **(A)** The morphology of parental LNCaP cells, LNCaP cells after 14 days ADT or 65 days ADT and 10 µM enzalutamide treatment is depicted. After 14 days neurite-like cell processes can be observed, while after 65 days the cells show a star-like appearance (Magnification: 100X, scale bar: 100 µm). **(B)** To determine the size and granularity of the cells, the forward scatter (FSC, size) and sideward scatter (SSC, granularity) were measured by flow cytometry. The size (FSC, p < 0.0001) and granularity (SSC, p < 0.0001) are significantly decreased for enzalutamide-resistant cells (LNCaP_EnzR_, dark) compared with parental LNCaP (light) cells. Box plots depict the median and SD of four independently performed experiments (****p < 0.0001). The histograms show representative flowcytometry results. **(C)** QRT-PCR analyses of prostate and neuroendocrine marker gene expression in parental and enzalutamide-resistant LNCaP cells were performed to validate the neuroendocrine phenotype of LNCaP_EnzR_ cells. The expression of prostate markers AR and PSA (p < 0.0001) is significantly decreased whereas expression of neuroendocrine markers CHGA, SYP (p<0.0001) and NSE (p=0.0002) is increased in LNCaP_EnzR_ cells. Graphs show the mean and SD from four independently performed experiments (***p < 0.001; ****p < 0.0001).

The role of EREG during different stages of PCa progression was analyzed using five cell lines with different characteristics. The CSPC cell line LNCaP represents adenocarcinoma, 22Rv1 cells express an AR splice variant, DU145 as well as PC3 cells are AR negative but express other steroid hormone receptors and different oncogenic signaling pathways are activated. Therefore, 22Rv1, DU145 and PC3 cells are castration-resistant cell lines (22). To establish a stable treatment-induced neuroendocrine prostate cancer (tNEPC) *in vitro* model, the previously described neuroendocrine differentiation (NED) *in vitro* model (8) was advanced. The morphological alterations apparent during treatment of the LNCaP cells were photographically documented and are depicted in **Figure 1A**. The neurite-like processes are typical for LNCaP cells after 14 days of androgen deprivation, but they disappear after 65 days. A further morphological characteristic of LNCaP_EnzR_ cells is the significant alteration in size (left graph, p<0.0001) and granularity (right graph, p<0.0001) (**Figure 1B**). The LNCaP_EnzR_ are 10% smaller in diameter and 35% less granulated than parental LNCaP cells determined by flow cytometer analysis using forward and sideward scatter. Additionally, several marker genes, which allow conclusions of the lineage, were analyzed by qRT-PCR. **Figure 1C** reveals that luminal prostate specific marker genes *AR* and *PSA* (p<0.0001) are significantly decreased while clinical neuroendocrine marker genes *CHGA*, *SYP* (p<0.0001) and *NSE* (p=0.0002) are significantly increased. Summarized, LNCaP_EnzR_ can be used as a model to study tNEPC *in vitro*.

### 3.2 Epiregulin biosynthesis and secretion is elevated in CRPC cells

**Figure 2:**
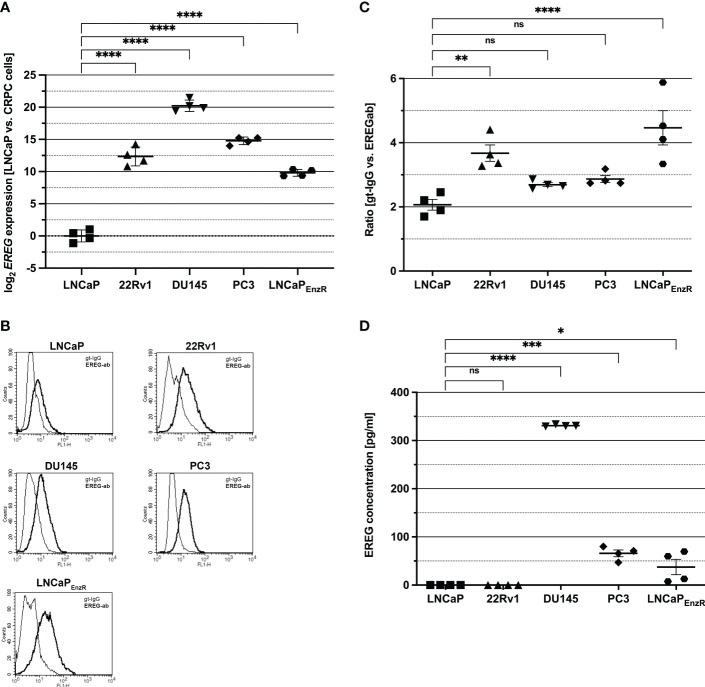
EREG expression and secretion is higher in CRPC in comparison to LNCaP cells (CSPC). **(A)** The expression of EREG after ADT treatment was assumed to be increased according to preliminary data. Analysis of EREG expression using qRT-PCR show an increased (p < 0.0001) EREG expression in CRPC cells in comparison to castration sensitive LNCaP cells. **(B)** Flow cytometry analysis shows the presence of EREG (dark) on the cell surface of CSPC and CRPC cell lines. Goat-IgG (light) is used as a control. Histograms show representative flow cytometry results for CSPC and CRPC cell lines using EREG antibody and goat-IgG. **(C)** The ratio is determined from the mean of the fluorescence intensity of the sample compared to the control. EREG protein on cell surface of 22Rv1 (p=0.0043) and LNCaP_EnzR_ (p < 0.0001) cells is significantly increased in comparison to CSPC cells whereas EREG protein on DU145 (p=0.55) and PC3 (p=0.29) is slightly, but not significant increased compared to CSPC cells. **(D)** PCa cell culture supernatant was collected and EREG protein was investigated by sandwich ELISA. Whereas LNCaP and 22Rv1 cells secrete no EREG protein, it was detected in DU145 (330 pg/ml), PC3 (66 pg/ml) and LNCaP_EnzR_ (37 pg/ml) supernatant. All graphs show the mean and SD from four independently performed experiments (ns, not significant; *p < 0.05; **p < 0.01; ***p < 0.001; ****p < 0.0001).

To investigate *EREG* expression in different PCa cell lines, qRT-PCRs were performed and normalized to expression in LNCaP cells (**Figure 2A**). Whereas LNCaP cells show almost no *EREG* expression, in CRPC cell lines expression is (p<0.0001) increased (1 x 10^3^ – 1.3 x 10^4^-fold). DU145 cells exhibit the highest *EREG* expression which is 1.05 x 10^6^-fold enhanced compared to LNCaP cells. Due to the localization of proepiregulin in the plasma membrane, a specific antibody staining and flow cytometry analysis were used to determine proepiregulin amount. **Figures 2B, C** depict the presence of proepiregulin on the cell surface of all PCa cell lines, but in comparison to LNCaP cells, 22Rv1 (p=0.0043) and LNCaP_EnzR_ (p<0.0001) cells contain more proepiregulin on the cell surface. ELISA was used to determine the EREG concentration in the supernatant (**Figure 2D**). The results show that no EREG is secreted by LNCaP and 22Rv1 cells. In contrast, DU145 (p<0.0001), PC3 (p<0.0001) and LNCaP_EnzR_ (p=0.027) cells secrete EREG, whereby DU145 cells secrete the highest amount with about 330 pg/ml of mature EREG. These results indicate a correlation between aggressiveness of PCa cell lines and elevated EREG secretion.

### 3.3 Epiregulin presence correlates with increasing aggressiveness of PCa samples

**Figure 3:**
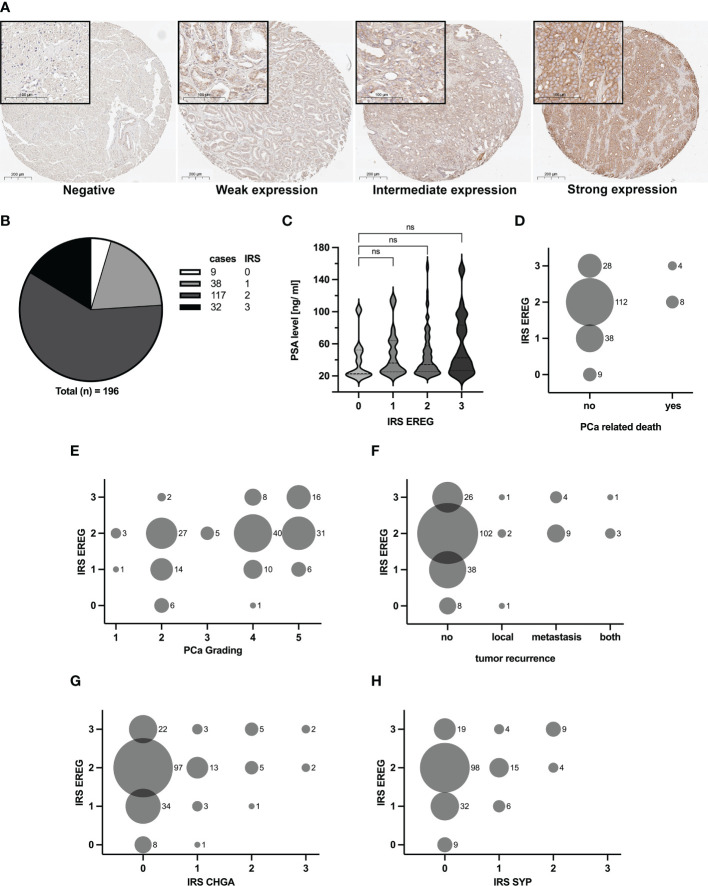
Amount of EREG correlates with increasing aggressiveness of PCa samples. TMA with 196 samples were immunohistochemically stained with EREG antibody. **(A)** Representative overview and detail pictures of EREG staining in PCa samples for different categories of IRS (Overview pictures: Magnification 200x, scale bar 200 µm; Detail pictures in black boxes: Magnification 630x, scale bar 100 µm). Samples with an IRS of 0 exhibit no expression of EREG, while samples with an IRS of 1 show a weak EREG expression. An IRS of 2 indicates an intermediate EREG expression and an IRS of 3 a strong expression. **(B)** The pie chart shows the distribution of cases split by IRS for EREG. **(C)** Violin plot with correlation of EREG IRS and preoperative PSA levels (median: dashed line, 95% quartile: dotted line). The EREG IRS tends to correlate positively with PSA level without significance (p=0.29; ns, not significant). **(D–H)** Bubble plots show the distribution of EREG IRS in relation to **(D)** PCa related death, **(E)** PCa grading, **(F)** tumor recurrence, **(G)** CHGA IRS and **(H)** SYP IRS. The size of the bubbles indicates the relative number of cases, while the numbers to the right of the bubbles represent the absolute number of cases. CHGA and SYP IRS were determined in the same manner as for EREG.

Human skin and kidney tissues were used to establish the EREG staining, whereas unspecific goat-IgG served as a control. **Supplemental Figure 1** shows the specific EREG staining of epithelial cells in the skin and kidney tubule. Subsequently, TMAs were stained, immunoreactive score (IRS) according to Remmele (20) were determined and the results were classified into four categories depending on the IRS. **Figure 3A** shows the four different categories, negative, weak, intermediate and strong expression. The distribution of cases after evaluation is depicted in **Figure 3B**. Nine cases showed an IRS of zero and they were defined as negative. 38 cases were rated with an IRS of one and 117 cases were rated with an IRS of two. These cases were classified as weak and intermediate expression, respectively. A strong expression of EREG, i.e. an IRS of three, was identified in 32 cases. Subsequently, specific patient data was correlated with corresponding EREG IRS. **Figure 3C** shows a correlation of the serum PSA value with EREG IRS. Although no significant difference is observed, it seems that a high serum PSA value is tended (p=0.29) to correlates with a higher EREG IRS. In the following, the aggressiveness of PCa was compared with the EREG IRS. First, a correlation between EREG IRS and PCa-related death of patients was performed (**Figure 3D**). PCa-related death of patients occurred only with intermediate or strong EREG expression. Histologically, the aggressiveness of PCa is determined by grading, which is based on the Gleason score. **Figure 3E** shows a positive correlation between rising PCa grade and high EREG IRS, but high EREG IRS does not automatically cause a high PCa grading. In addition to the aggressiveness and stage of the carcinoma, tumor recurrence is also crucial for continuing patient treatment. Tumor recurrence was divided depending on its location into local, metastatic, or local and metastatic (both). With one exception, tumor recurrence was only associated with intermediate or high EREG expression (**Figure 3F**). Finally, we examined whether EREG expression is related to NED. The IRS for the clinical NE markers chromogranin A (CHGA) and synaptophysin (SYP) were determined as described previously and correlated with the EREG IRS. Independent of CHGA (**Figure 3G**) or SYP (**Figure 3H**) IRS, differently pronounced EREG expressions were detected. However, samples with a CHGA or SYP IRS above two are always associated with a high EREG IRS. In summary, the results suggest enhanced expression of EREG in advanced metastatic prostate carcinomas.

### 3.4 Elevated activation of SMAD2/3 in CRPC cells lead to enhanced *EREG* transcription and secretion

**Figure 4:**
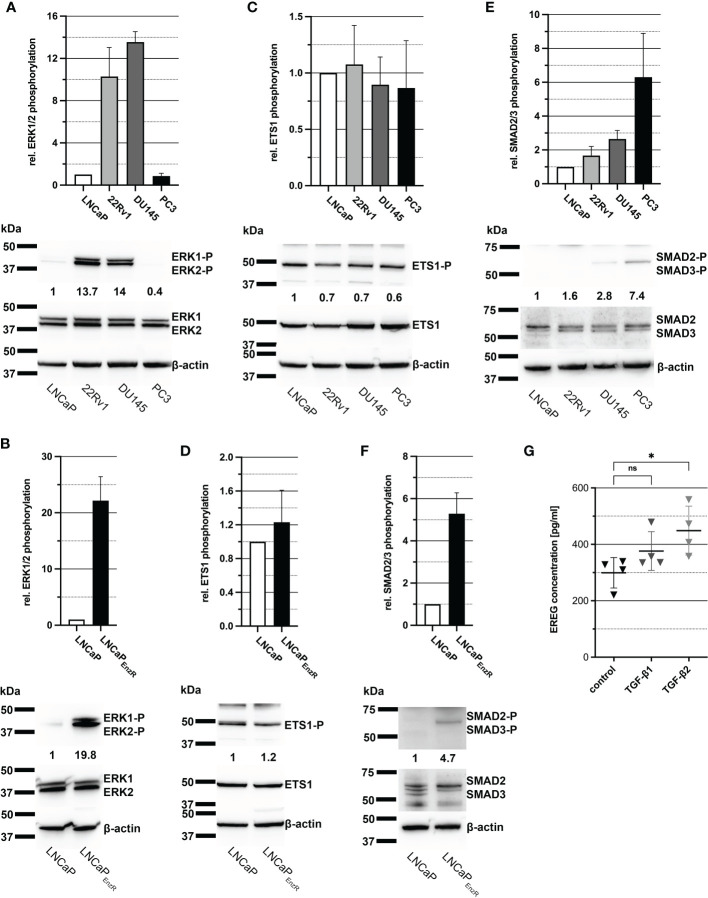
Elevated phosphorylation of SMAD2/3 seems to enhance EREG expression in PCa cells. **(A)** Column graph shows ERK1/2 phosphorylation at Thr202/Tyr204 in PCa cell lines LNCaP, 22Rv1, DU145 and PC3. ERK1/2 phosphorylation in 22Rv1 and DU145 cells are increased in comparison to LNCaP cells. Additionally, a representative immunoblot image for ERK1/2 phosphorylation in PCa cell lines is illustrated. **(B)** ERK1/2 phosphorylation at Thr202/Tyr204 of LNCaP in comparison to LNCaP_EnzR_ cells and a representative immunoblot image are shown. Phosphorylation of ERK1/2 is enhanced in LNCaP_EnzR_ cells. **(C)** The column graph shows the phosphorylation of transcription factor ETS1 at Thr38 in the different PCa cell lines without differences in ETS1 phosphorylation and expression. The adjacent representative immunoblot illustrates the result. **(D)** As revealed in the graph and the representative immunoblot, LNCaP_EnzR_ exhibit no alteration in ETS1 phosphorylation at Thr38 compared with the parental LNCaP cells as well. **(E)** The bar graph and representative immunoblots shows the phosphorylation of transcription factor SMAD2/3 at Ser465, Ser467, Ser423, Ser425 in PCa cell lines. Compared to LNCaP cells, SMAD2/3 phosphorylation in 22Rv1 and DU145 is slightly increased, but PC3 cells show a noticeably higher phosphorylation of SMAD2/3. **(F)** SMAD2/3 phosphorylation of LNCaP_EnzR_ cells is illustrated in a column graph and a representative immunoblot image. Phosphorylation in SMAD2/3 at Ser465, Ser467, Ser423, Ser425 is increased in LNCaP_EnzR_ in comparison to parental LNCaP cells. All graphs show the mean and SD from four independently performed experiments. **(G)** Total EREG protein in cell culture supernatant of DU145 cells after TGF-β1 (p=0.2649) or TGF-β2 (p=0.0283) treatment is significantly elevated in comparison to untreated DU145 cells. All graphs show the mean and SD from four independent experiments (ns, not significant; *p < 0.05).

As previously shown in **Figure 2A**, *EREG* expression is increased in CRPC cells in comparison to CSPC cells and it is assumed to be caused by the elevated activity of some signaling pathways and transcription factors. Activity of MAPK signaling pathway results i.a. in phosphorylation of transcription factor ETS-1, which binds to the *EREG* promotor and stimulates transcription (23). To determine the activity of MAPK pathway and ETS-1, immunoblots from cell lysates were performed, detecting phosphorylated amino acid residues, which lead to activation of the proteins. **Figure 4A** depicts a bar graph and one representative immunoblot image illustrating a 14-fold enhanced phosphorylation of ERK1/2 at Thr202/Tyr204 in 22Rv1 and DU145 cells. Additionally, LNCaP_EnzR_ cells exhibit an alomst 20-fold higher phosphorylation of ERK1/2 at Thr202/Tyr204 compared to parental LNCaP cells (**Figure 4B**). After demonstrating enhanced activity of the MAPK pathway, phosphorylation of ETS1 was examined whereat the phosphorylation of ETS1 is stable in all cell lines (**Figure 4C**). LNCaP_EnzR_ cells also show no alteration in ETS1 content or phosphorylation compared to parental LNCaP cells (**Figure 4D**). Another transcription factor stimulating *EREG* expression is SMAD2/3, which is activated *via* the TGF-β signaling pathway (24, 25). In addition, our recent study showed that ADT of LNCaP cells lead to increased expression of TGF-β2. **Supplemental Figure 2** depicts the 90-fold (p<0.0001) increased expression of TGF-β1 in LNCaP_EnzR_ cells compared to parental LNCaP cells. Immunoblot analysis of SMAD2/3 phosphorylation at Ser465, Ser467/Ser423, Ser425 revealed a slightly 1.5 - 2.5-fold increased phosphorylation in 22Rv1 and DU145 cells on average (**Figure 4E**), whereas PC3 and LNCaP_EnzR_ cells showed a stronger, 5 - 6-fold increase of SMAD2/3 phosphorylation compared to LNCaP cells (**Figures 4E, F**). These results suggest that the enhanced expression of *EREG* in some CRPC cell lines is caused by SMAD2/3 phosphorylation. To verify this hypothesis, DU145 cells were treated with 10 ng/ml TGF-β1 or TGF-β2 for 24 h. Subsequently, the EREG concentration in the cell culture supernatant was determined by ELISA. After treatment of DU145 cells with TGF-β1 the amount of EREG in cell culture supernatant tend to increase, but the difference to untreated cells is not significant (p=0.2649). However, the treatment of DU145 cells with TGF-β2 results in a significant (p=0.0283) increase of EREG protein in cell culture supernatant (**Figure 4G**). The data indicate an effect of the TGF-β signaling pathway on EREG transcription and secretion.

### 3.5 MiRNAs are involved in post-transcriptionally regulation of EREG expression

**Figure 5:**
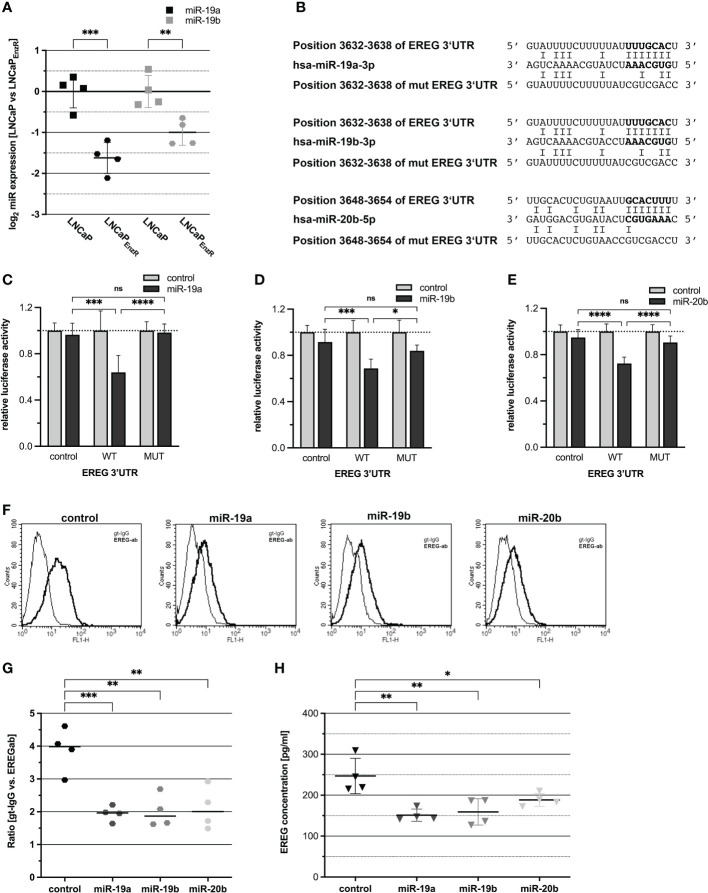
EREG protein biosynthesis is post-transcriptionally regulated by miRNAs. **(A)** The expression of miR-19a and -19b that were assumed to be reduced according to preliminary data was assessed by qRT-PCR. MiR-19a (p=0.0001) and -19b (p=0.0053) were significantly reduced in LNCaP_EnzR_ compared to parental LNCaP cells. **(B)** Predicted miRNA binding sites in the 3’UTR of EREG mRNA for miR-19a, -19b, -20b and the mutated miRNA binding sites are shown. The bold nucleotides in the sequences represent the miRNAs seed sequence and its corresponding binding sites. **(C–E)** For luciferase reporter assay, miRNA expression plasmids (control grey bar, miRNA black bar) were cotransfected with empty reporter plasmid (control), reporter gene construct containing wildtype EREG 3’UTR (WT) or reporter gene construct containing mutated EREG 3’UTR (MUT). The luciferase activity of the reporter gene plasmid coexpressed with control expression plasmid was set to 1. MiR-19a (p=0.0001), -19b (p=0.0006) and -20b (p<0.0001) significantly reduce luciferase activity of the reporter gene construct containing EREG 3’UTR in comparison to the control. After mutation of the seed sequence, the corresponding miRNAs are not able to reduce the luciferase activity. **(F)** Flow cytometry analysis using EREG antibody show the EREG presence on the cell surface of LNCaP_EnzR_ cells transfected with miRNA expression plasmid. The histograms show representative flow cytometry analyses of LNCaP_EnzR_ cells transfected with miRNA expression plasmid using EREG antibody (dark) and goat-IgG (light). **(G)** The ratio is determined from the mean of the fluorescence intensity of the specific EREG antibody compared to the control (goat-IgG). EREG protein on cell surface of LNCaP_EnzR_ cells transfected with expression plasmids for miR-19a (p=0.0008), miR-19b (p=0.001) or miR-20b (p=0.0015) is significantly decreased in comparison to LNCaP_EnzR_ cells transfected with control expression plasmid. All graphs show the mean and SD from four independent experiments (ns not significant; *p < 0.05; **p < 0.01; ***p < 0.001; ****p < 0.0001). **(H)** Total EREG protein in cell culture supernatant of DU145 cells transfected with expression plasmids for miR-19a (p=0.0015), miR-19b (p=0.0029) or miR-20b (p=0.038) is significantly decreased in comparison to DU145 cells transfected with control expression plasmid. All graphs show the mean and SD from four independent experiments (ns, not significant; *p < 0.05; **p < 0.01).

After transcription, the EREG mRNA is translated into proepiregulin. Translation is regulated by multiple mechanisms, including translational inhibition by miRNA binding to specific sequences in the 3’-untranslated region (3’UTR) of mRNAs. Additionally, several studies have demonstrated a role of miRNAs in the progression of PCa (26, 27). To investigate the regulation of EREG by miRNAs, an *in silico* analysis of the EREG 3’UTR was performed using the “TargetScan 7.2” website (21). Within the EREG 3’UTR, several putative binding sites for miRNAs were predicted. Subsequently, miRNAs were selected for further investigation which putatively bind to the EREG 3’UTR and which are repressed in LNCaP_EnzR_ cells. **Figure 5A** shows a 0.6-fold decrease (p=0.0001) of miR-19a expression and 0.4-fold decrease (p=0.0053) of miR-19b expression in LNCaP_EnzR_ cells. The decreased expression of miR-20b after NED of LNCaP cells has been confirmed previously (8). Furthermore, in addition to the miRNA sequences, the binding sites within the EREG 3’UTR and the mutated binding sites, are shown in **Figure 5B**. Dual-luciferase reporter gene assays were performed to investigate the binding of miRNAs to the *EREG* 3’UTR, but previously, the increased expression of miRNAs after transfection with expression plasmid was confirmed by qRT-PCR (**Supplementary Figure 3**). **Figures 5C–E** demonstrate the relative luciferase activity after co-transfection of the reporter plasmids harboring the EREG 3’UTR or empty control with the miRNA expression plasmids for miR-19a (5C), miR-19b (5D), and miR-20b (5E). Increased expression of miR-19a (p=0.0001), miR-19b (p=0.0006), and miR-20b (p<0.0001) caused a significant 30-40% reduction of relative luciferase activity. To verify an interaction between miR-19a, -19b, or -20b and EREG 3’UTR, the seed-sequence inside the binding site in the EREG 3’UTR was specifically mutated and dual-luciferase reporter gene assays were performed. Inhibition of luciferase activity by enhanced expression of miR-19a, miR-19b, and miR-20b was abolished after mutation of the corresponding binding site. The influence of miRNAs on endogenous proepiregulin synthesis was determined by flow cytometry. LNCaP_EnzR_ cells were transfected with miRNA expression plasmids, labeled with an EREG antibody and analyzed by flow cytometry. The representative histograms as well as the dot plot (**Figures 5F, G**) show a significant 50% decrease of proepiregulin on the cell surface after induced miR-19a (p=0.0008), miR-19b (p=0.001) or miR-20b (p=0.0015) expression. The flow cytometry analysis reveals a negative regulation of proepiregulin synthesis mediated by miR-19a, miR-19b, and miR-20b. To investigate the effect of miRNAs on EREG secretion, DU145 cells were transfected with the miRNA expression plasmids. After 24 h ELISAs were performed to determine the EREG content in cell culture supernatant of transfected cells. DU145 cells were used because this cell line previously showed the highest endogenous EREG amount in cell culture supernatant. **Figure 5H** shows that enhanced expression of miR-19a (p=0.0015), miR-19b (p=0.0029) and miR-20b (p=0.038) significantly decreased the EREG content in the cell culture supernatant of DU145 cells. In summary, these data provide evidence for posttranscriptional regulation of EREG by miR-19a, miR-19b, and miR-20b.

### 3.6 Proepiregulin-shedding proteases are increased in CRPC compared to CSPC cells

**Figure 6:**
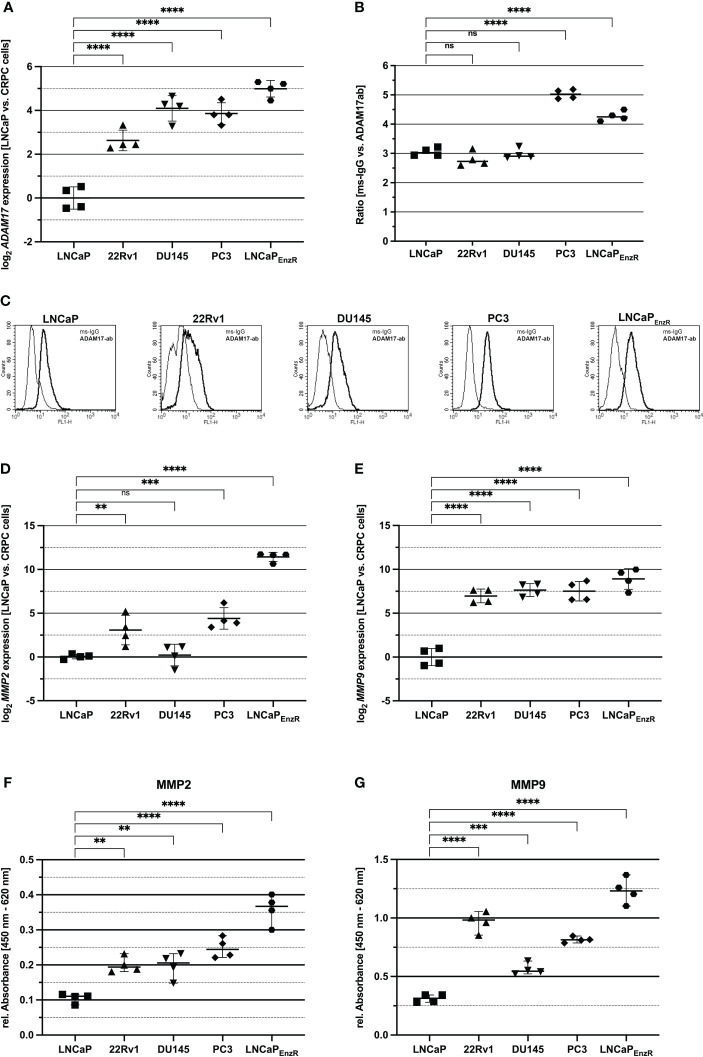
Proteases, shedding the ectodomain of EREG are increased in CRPC compared to CSPC cells. **(A)** The dot plot depicts the ADAM17 expression of CSPC and CRPC cell lines using qRT-PCR analysis. 22Rv1, DU145, PC3 and LNCaP_EnzR_ (p<0.0001) significantly express more ADAM17 compared to LNCaP cells. **(B)** Flow cytometry analysis using ADAM17 antibody show the ADAM17 presence on the cell surface of CSPC and CRPC cell lines. ADAM17 protein level on cell surface of PC3 (p<0.0001) and LNCaP_EnzR_ (p < 0.0001) cells is increased in comparison to LNCaP cells whereas ADAM17 protein content on 22Rv1 and DU145 is not altered. **(C)** The pictures show representative flow cytometry analysis of CSPC and CRPC cell lines using ADAM17 antibody (dark) and mouse-IgG (light) as a control. MMP2 **(D)** and MMP9 **(E)** expression in CSPC and CRPC cell lines was determined by qRT-PCR. In comparison to LNCaP cells 22Rv1 (p=0.0057), PC3 (p=0.0002) and LNCaP_EnzR_ (p < 0.0001) cells show an increased expression of MMP2. The MMP9 expression of 22Rv1 (p < 0.0001), DU145 (p < 0.0001), PC3 (p < 0.0001) and LNCaP_EnzR_ (p < 0.0001) is enhanced compared to LNCaP cells. **(F)** Solid phase ELISA using a specific MMP2 antibody demonstrates the secretion of MMP2 in cell culture supernatant of PCa cell lines. The ELISA results reveal that MMP2 is secreted by all five PCa cell lines, but 22Rv1 (p=0.0021), DU145 (p=0.0026), PC3 (p < 0.0001) and LNCaP_EnzR_ (p < 0.0001) cells secrete a higher amount of MMP2 protein in comparison to LNCaP cells. **(G)** PCa cell culture supernatant was collected and MMP9 protein was investigated by solid phase ELISA. All PCa cells secrete MMP9 protein, but the CRPC cell lines 22Rv1 (p < 0.0001), DU145 (p=0.0005), PC3 (p < 0.0001) and LNCaP_EnzR_ (p < 0.0001) secrete more MMP9 protein than CSPC cell line LNCaP. All graphs show the mean and SD from four independent experiments (ns, not significant; **p < 0.01; ***p < 0.001; ****p < 0.0001).

Proepiregulin is transported to the cell membrane, where ADAM17 is located and cleaves the EGF-like domain of proepiregulin whereby mature EREG is secreted into the extracellular space (10, 12). To analyze whether *ADAM17* is expressed in the different cell lines, qRT-PCRs were performed. **Figure 6A** shows a significant (p<0.0001) 2.5-5-fold increased *ADAM17* expression in all CRPC cell lines compared to parental LNCaP cells. Flow cytometry analysis was used to analyze existence of ADAM17 on the cell surface. **Figures 6B, C** reveal the presence of ADAM17 on the cell surface of all PCa cell lines, but in comparison to LNCaP, PC3 (p<0.0001) and LNCaP_EnzR_ (p<0.0001) cells contain significantly more ADAM17 on the cell surface. Two additional extra cellular proteases (MMP2 and MMP9) are involved in ectodomain shedding of proepiregulin (11). The *MMP2* and *MMP9* expression in PCa cell lines was examined using qRT-PCRs. **Figure 6D** shows, in comparison to LNCaP cells, a significantly elevated *MMP2* expression in 22Rv1 (p<0.0001), PC3 (p<0.0001) and LNCaP_EnzR_ (p<0.0001) cells. The *MMP9* expression in 22Rv1 (p<0.0001), DU145 (p<0.0001), PC3 (p<0.0001) and LNCaP_EnzR_ (p<0.0001) is also significantly, 128-fold, increased compared to LNCaP cells (**Figure 6E**). Subsequently, cell culture supernatant was collected and ELISA were performed to study MMP2 and MMP9 content in supernatant of PCa cell lines. Due to a lacking standard for MMPs, the relative absorbance was calculated. **Figure 6F** demonstrates the presence of MMP2 in supernatant of all PCa cell lines. The relative absorbance in supernatant derived from 22Rv1 (p=0.0021), DU145 (p=0.0026), PC3 (p<0.0001) and LNCaP_EnzR_ (p<0.0001) cells were 2- to 3-fold elevated compared to LNCaP cells. Furthermore, all PCa cells secrete MMP9 protein (**Figure 6G**), whereat CRPC cell lines 22Rv1 (p<0.0001), DU145 (p=0.0005), PC3 (p<0.0001) and LNCaP_EnzR_ (p<p 0.0001) secrete significantly more MMP9 protein than LNCaP cells. These results suggest a higher ADAM17 presence on cell surface as well as MMP2 and MMP9 secretion of CRPC cells leading to elevated EREG secretion.

## 4 Discussion

Treatment success of prostate cancer is highly dependent on the classified histology and individual molecular characteristics. Therefore, it is important to identify accessible markers that provide reliable information on tumor progression and new therapeutic targets to improve individual treatments. CRPC cells often exhibit therapy-resistance but also increased secretion of growth factors and hormones stimulating dedifferentiation, proliferation, and survival of other tumor cells (5, 6). One of these growth factors might be the EGFR ligand EREG, activating important oncological signaling pathways. EREG is usually overexpressed in several human cancers including bladder, brain, breast, colorectal and lung cancer and it is used as a predictive biomarker i.e. in metastatic colorectal cancer (13, 28). The relevance of EREG as an oncogene in many different entities and the increased expression after ADT in PCa cells, suggest EREG as an interesting candidate gene for further characterization in different stages of PCa.

Initially, a tNEPC cell line was established. This method is well known and several studies demonstrate that androgen-dependent cells developing castration-resistance by ADT and enzalutamide treatment are associated with a gain of neuroendocrine features (29–31). The cells show morphological alterations and genetic neuroendocrine characteristics. Concluded, LNCaP_EnzR_ cell line is a feasible *in vitro* model for tNEPC. Accordingly, five cell lines representing different stages and characteristics of PCa were used in this study to investigate the relevance of EREG in PCa. Here, the results of Torring (14, 15) and Dankert (8) could be extended demonstrating that not only ADT of CSPC cells leads to an increased *EREG* expression, moreover, all four CRPC cell lines exhibit a higher *EREG* expression than LNCaP cells. While *EREG* expression was the highest in DU145 cells, also confirmed by Carrión-Salip and colleagues (32), increased proepiregulin content on cell surface was rather detected in 22Rv1 and LNCaP_EnzR_ cells. To investigate whether CRPC cells secrete EREG and affect surrounding or distant cells, the EREG content in the cell culture supernatant was determined. Highest amounts of EREG was detected in DU145 supernatant indicating rapid cleavage of proepiregulin by proteases. Taken together, our data indicate that the expression and secretion of EREG is a mechanism of CRPC cells to gain resistance to androgen blockade. To underline the relevance of EREG in different stages of PCa, a TMA was stained with an EREG antibody. The data suggest an accordance of high EREG expression and tumor recurrence, metastasis, increased PCa grading and PCa-related death. However, the number of 12 PCa-related deaths is small and should be considered with caution. Furthermore, another study shows an association of increased *EREG* transcription with resistance to anti-proliferative agent metformin (33), supporting the assumption, that resistance to different therapeutics is caused or supported by increased secretion of EREG leading to activation of the EGFR signaling pathway. Further studies demonstrated that the activation of the EGFR signaling pathway in turn leads to increased EGFR ligand expression, resulting in a positive feedback loop (34, 35). Consequently, a permanently high level of EGFR ligands could be maintained.

After demonstrating an enhanced EREG secretion by CRPC cells, mechanisms of EREG regulation should be identified. According to a study of Cho and colleagues, *EREG* transcription is regulated by the activity of MAPK signaling pathway. A downstream target of the MAPK pathway is the transcription factor ETS1, which is directly involved in *EREG* expression (23). Furthermore, ETS1 expression is increased in high-grade PCa and elevated expression as well as transcriptional activity promotes an aggressive and castration-resistance in PCa cells (36). Although, the results show that the MAPK signaling pathway is activated in 22Rv1, DU145, and LNCaP_EnzR_ cells, the phosphorylation of ETS1 is not altered compared to CSPC cells. Another transcription factor SMAD2/3, a downstream target of TGFβR also induces expression of *EREG* (24, 25) shows increased phosphorylation in DU145, PC3, and LNCaP_EnzR_ cells. TGF-β pathway inhibits proliferation and promotes apoptosis in epithelial, luminal prostate cells, but switches to an oncogene in advanced PCa, facilitating PCa progression to metastasis (37). In this study, treatment of DU145 cells, a metastatic PCa cell line with TGF-β resulted in increased secretion of EREG. This in turn could result in increased activity of other oncogenic signaling pathways, which drive further tumor progression. However, elevated *EREG* expression could also be caused by other transcription factors. In BC1 or Caco-2 cells, EREG expression and secretion is increased after addition of IL-6, IL-17 or IL-1β (38, 39). The addition of these cytokines activates NFκB and STAT3, which may cause the increased *EREG* expression. Moreover, epigenetics may be involved in EREG expression by modulating the binding of transcription factors (40).

After transcription, further regulatory options of EREG biosynthesis are observed. One of these concerns the binding of miRNAs to the 3’UTR of *EREG* mRNA leading to decreased translation. In this study, a direct binding of miR-19a, miR-19b, and miR-20b to the 3’UTR of *EREG* mRNA could be demonstrated. Furthermore, a diminished EREG protein content on the cell surface and in cell culture supernatant after miRNA over expression confirm an effect of these miRNAs on endogenous EREG protein. Consequently, miR-19a, miR-19b, and miR-20b are involved in the post transcriptionally regulation of EREG. MiR-19a/b are members of the miR-17-92a cluster. Whereas some studies showed a miR-19a/b overexpression in PCa and decreased invasiveness after miR-19a/b deletion in mice, other studies showed that expression of miR-17-92a cluster is reduced in cancerous prostate tissues and decreased activation of oncogenic pathways (41, 42). Similarly, miR-20b, a member of the miR-106b-25 cluster cannot be classified as either an oncogene or tumor suppressor gene. Guo and colleagues showed that miR-20b promotes proliferation and migration by directly regulating PTEN in PC3 cells. In contrast, miR-20b overexpression in LNCaP cells results in decreased cell growth, colony formation and increased apoptosis. Therefore, a different role of miR-20b is suggested depending on the aggressiveness of PCa cell line (8, 43). In summary, the exact role of miR-19a/b and miR-20b in prostate cancer has not been clarified to date. A role as both oncogene and tumor suppressor seems possible and depends on the stage of PCa. Several other studies already demonstrated a contribution of miRNAs in the post-transcriptional regulation of epiregulin. Siu and colleagues showed that autocrine-activated EREG expression is associated with repressed miR-203 which directly binds to the 3’UTR of EREG and regulates the stability of mRNA in DU145 cells (44). Interestingly, whereas EREG expression was elevated, miR-203 were repressed in androgen depleted LNCaP cells (8).

Before mature EREG is released, it is localized as proepiregulin in the plasma membrane. This is the last cellular regulation site for EREG secretion. Proteases such as ADAM17, MMP2 and MMP9 are responsible for shedding the ectodomain of proepiregulin (10–12). Here, the presence of ADAM17 was detected on the cell surface of all PCa cell lines, with PC3 and LNCaP_EnzR_ cells showing the highest ADAM17 levels. In accordance, Karan and colleagues revealed a strong expression of ADAM17 in PCa cell lines and patient samples (45). Overexpression of ADAM17 in PCa cells leads to enhanced cell proliferation, invasiveness as well as EGFR/AKT and EGFR/MAPK signaling activity. Additionally, matrix metalloproteases MMP2 and MMP9 are also regulated by ADAM17 (46). In this study, both matrix metalloproteases could be detected, especially in the cell culture supernatant of CRPC cells. In line with this, the MMP activity increases during PCa progression and it contributes to neuroendocrine prostate carcinogenesis, metastasis, and angiogenesis (47). Interestingly, expression and secretion of MMP2 and MMP9 is stimulated in prostatic epithelial cells treated with TGF-β (48), whose expression is also increased in LNCaP_EnzR_ cells. In addition to enhanced SMAD2/3 phosphorylation, TGF-β could also enhance the expression of MMP2 and MMP9, suggesting that it stimulates EREG secretion at multiple sites.

## 5 Conclusion

Summarized, this study indicates that EREG plays a crucial role during PCa progression and developing castration-resistance. EREG expression seems to correlate with tumor recurrence, metastasis and increased grading of PCa patient samples. Furthermore, this study demonstrates the increased amount and activity of EREG biosynthesis machinery in CRPC cell lines. In conclusion, EREG could be a diagnostic tool to detect a developing resistance, PCa metastases or tumor recurrence and subsequently enable personalized therapies of patients. Consequently, investigations should be performed, whether EREG content is specifically increased in blood, urine or ejaculate derived from patients with CRPC. In addition, EREG could also be a therapeutic target specifically for mCRPC. EGFR inhibitors are abundant but have not exhibited the expected success in PCa therapy (49). Potentially, identifying patients with EREG deregulation could select patients who would benefit from targeting therapy against EREG or EGFR. Another option is to use antibodies directly targeting EREG and block epiregulin-mediated EGFR signaling (50). However, sufficient studies are still lacking to confirm the effect of antibodies directed against epiregulin.

## Data availability statement

The original contributions presented in the study are included in the article/**Supplementary Material**. Further inquiries can be directed to the corresponding author.

## Ethics statement

The studies involving human participants were reviewed and approved by KEK Bern no. 128/2015. The patients/participants provided their written informed consent to participate in this study.

## Author contributions

Conception, MW. Methodology, MW and JD. Validation, MW and BR. Formal analysis, MW. Investigation, MW, BR, CW, and EC. Resources, MS and MK. Data curation, MW. Writing - original draft preparation, MW. Writing - review and editing, MW, JD, CW, BR, EC, MS, MK, and GW. Visualization, MW. Supervision, MW, JD, and GW. Project administration, MW, JD, and GW. Acquisition, GW. All authors contributed to the article and approved the submitted version.
