# Supplementary material for: Epiregulin expression and secretion is increased in castration-resistant prostate cancer
Source: Front Oncol. 2023 Mar 13;13:1107021. doi: 10.3389/fonc.2023.1107021 (PMC10040687; doi:10.3389/fonc.2023.1107021)
Supplement: Supplementary file 1 [file DataSheet_1.pdf]

Supplementary Information

Enzyme-linked Immunosorbent Assay (ELISA) procedure in detail

Cells were seeded 90% confluent and cultured for 24 h in RPMI medium without additives. The supernatant was collected and centrifuged. Subsequently, the supernatant was concentrated 10-fold using Vivaspin® Turbo 15 (3 kDa MWCO) Centrifugal Concentrator (Sartorius, Goettingen, Germany). The EREG content in the cell culture supernatant was determined by sandwich ELISA using the Human Epiregulin DuoSet ELISA (R&D Systems, Minneapolis, Minnesota, US) according to the manufacturer's instructions. The protein content of MMP2 and MMP9 in the cell culture supernatant was determined by solid phase ELISA. Supernatant was added to an ELISA plate and incubated at 4 °C overnight. Next day, the plate was washed three times with 0.05% Tween® 20 in PBS and blocked with 1% BSA in PBS for 1 h at room temperature. In the following, after each step, the plate was washed three times. The signal determination and amplification occurred using a α-MMP2 (mouse, 2C1-1D12, Thermo Fisher Scientific, Schwerte, Germany) or α-MMP9 (mouse, 5G3, Thermo Fisher Scientific, Schwerte, Germany) antibody, a biotin-coupled α-mouse (goat, polyclonal, Jackson ImmunoResearch, Cambridgeshire, UK) antibody and streptavidin-coupled HRP (R&D Systems, Minneapolis, Minnesota, US). The antibodies and streptavidin-coupled HRP were dissolved in 1% BSA in PBS. ChromPure mouse IgG (Jackson ImmunoResearch, Cambridgeshire, UK) was used as a negative control. Incubation with antibodies occurred for 2 h at room temperature, while incubation with HRP-coupled streptavidin lasted 20 min at room temperature. After a final washing, 100 µl of TMB X-tra (Kementec, Taastrup, Denmark) was added. The reaction was stopped after 20 min by addition of 100 µl H2SO4 (0.2 M). Absorbance was measured using a CLARIO starPlus microplate reader (BMG Labtech, Ortenberg, Germany). The relative absorbance was calculated by subtracting the corrected 450 nm absorbance of the blank (unconditioned medium) and the corrected 450 nm absorbance of the negative control (ms-IgG).

Supplementary Materials

Supplementary Table 1: Oligonucleotides sequences used for qRT-PCR

Gene	Sequence
AR	F: 5'-GGAACCTAAGATGAGTAATATG-3'
	R: 5'-ATGAGCCAGATTGTGTAA-3'
CHGA	F: 5'-CTTCCTCCATCCTATCCA-3'
	R: 5'-TCATACAGAATATGTTACAGTCA-3'
EREG	F: 5'-GGCTCAAGTGTCAATAAC-3'
	R: 5'-ATGTCCACCAGATAGATG-3'
GAPDH	F: 5'-CTCTGGTAAAGTGGATATTGT-3'
	R: 5'-GGTGGAATCATATTGGAACA-3'
MMP2	F: 5'-CAGCATTCTCACTCCTAC-3'
	R: 5'-TACAGTCAGCATCTATTCTTG-3'
MMP9	F: 5'-GGCAGATTCCAAACCTTT-3'
	R: 5'-GCAAGTCTTCCGAGTAGT-3'
NSE	F: 5'-CAATGTGGGGGATGAAGG-3'
	R: 5'-GTGTAGCCAGCCTTGTCGAT-3'
PSA	F: 5'-AGTGTTTCTTAAATGGTGTAA-3'
	R: 5'-CATCCTATCTGTGTCCTC-3'
SYP	F: 5'-GAGTGATATGGCTTGTAG-3'
	R: 5'-TCTGTCCTCCTATTAACC-3'
TACE/ADAM17	F: 5'-TTATTGGTGGTAGCAGAT-3'
	R: 5'-AAGTGTTCCGATAGATGT-3'
TGF-β	F: 5'-GAACTCATTCAGTCACCAT-3'
	R: 5'-GCTTGTTTCCTCACCTTTA-3'
miR-19a-3P	F: 5'-TGTGCAAATCTATGCAAAACTGA-3'
	R: universal Primer (miScript II RT Kit, Qiagen, Hilden, Germany)
miR-19b-3P	F: 5'-TGTGCAAATCCATGCAAAACTGA-3'
	R: universal Primer (miScript II RT Kit, Qiagen, Hilden, Germany)

Supplementary Table 2: Antibodies used for immunodetection in Western Blot

Antibody	Host	Conjugate	Clone	Company
α-ERK1/2	rabbit	-	137F5	Cell Signaling, Frankfurt am Main, Germany
α-Phospho-ERK1/2 (Thr202/Tyr204)	rabbit	-	D13.14.4E	
α-ETS1	rabbit	-	JM92-32	Thermo Fisher Scientific, Schwerte, Germany
α-Phospho-ETS1 (Thr38)	rabbit	-	polyclonal	
α-SMAD2/3	rabbit	-	polyclonal	
α-Phospho-SMAD2/3 (Ser465, Ser467, Ser423, Ser425)	rabbit	-	polyclonal	
α-actin-β	mouse	HRP	AC15	Sigma-Aldrich, Hamburg, Germany
α-rabbit	goat	HRP	polyclonal	Jackson ImmunoResearch, Cambridgeshire, UK

Supplementary Table 3: Oligonucleotides sequences used for cloning and site directed mutagenesis

Application	Gene	Sequence
cloning	EREG 3'UTR	F: 5'-GGACTAGTCTCTAATCTCTCTGCCGAAAGTCA-3'
		R: 5'-CGAGCTCGAAAAACCGTTTACCAAAATGGCT-3'
	miR-19a-3P cds	F: 5'-CGGAATTCGCAGTGAAGGCACTTGTAGC-3'
		R: 5'-CGGGATCCCGAAGCTGGAGTTCTACAGC-3'
	miR-19b-3P cds	F: 5'-CGGAATTCGATGGTGGCCTGCTATTTCC-3'
		R: 5'-CGGGATCCGACACGCAACCCCAAAAGTG-3'
site directed mutagenesis	EREG 3'UTR mutation 1	F: 5'-GTATTTTCTTTTTATCGTCGACCCTGTAATTGCACTTT-3'
		R: 5'-AAAGTGCAATTACAGGGTCGACGATAAAAAGAAAATAC-3'
	EREG 3'UTR mutation 2	F: 5'-TTTGCACTCTGTAACCGTCGACCTTAAGTTTGAAGAGC-3'
		R: 5'-GCTCTTCAAACTTAAGGTCGACGGTTACAGAGTGCAAA-3'

Abbreviations: cds, coding sequence

Supplementary Figures

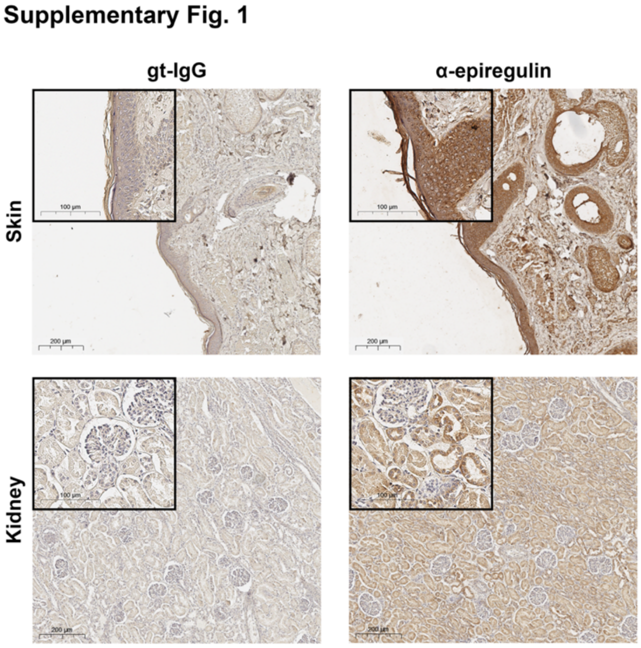

Supplementary Fig. 1 Establishing immunohistochemical (IHC) staining with an EREG antibody. Representative overview and detail pictures (black box) of skin and kidney using an EREG antibody served as positive controls. For negative control, goat (gt)-IgG was used in IHC staining instead of a specific EREG antibody (Overview pictures: Magnification: 200x, scale bar: 200 µm; Detail pictures in black boxes: Magnification: 630x, scale bar: 100 µm).

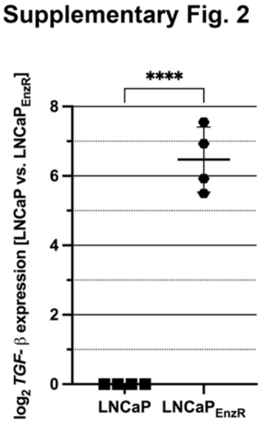

Supplementary Fig. 2 TGF-β, which leads to phosphorylation of SMAD2/3 is increased in $LNCaP_{EnzR}$. The dot plot depicts the TGF-β expression of $LNCaP_{EnzR}$ cells and LNCaP cells using qRT-PCR analyses. $LNCaP_{EnzR}$ ($p<0.0001$) express more TGF-β compared to parental LNCaP cells. Graph shows the mean and SD from four independent experiments (**** $p<0.0001$).

Supplementary Fig. 3

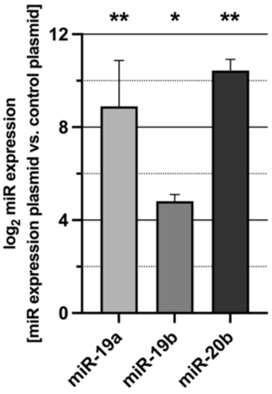

Supplementary Fig. 3 Validation of induction of miR-19a and -19b after transfection with the corresponding expression plasmids. The column diagram depicts the miRNA expression in HEK293T cells transfected with miRNA expression or control plasmid evaluated by qRT-PCR analysis. HEK293T cells express more miR-19a (p=0.0022), miR-19b (p=0.0211) or miR-20b (p=0.0012) after transfection with miRNA expression plasmid compared to cells transfected with control plasmid. Graph shows the mean and SD of two independent experiments (* p<0.05; ** p<0.01).
